# *ZmCIPK21*, A Maize CBL-Interacting Kinase, Enhances Salt Stress Tolerance in *Arabidopsis thaliana*

**DOI:** 10.3390/ijms150814819

**Published:** 2014-08-22

**Authors:** Xunji Chen, Quansheng Huang, Fan Zhang, Bo Wang, Jianhua Wang, Jun Zheng

**Affiliations:** 1 College of Agriculture and Biotechnology, China Agricultural University, No. 2 Yuanmingyuan West Road, Beijing 100094, China; E-Mails: chenxj713@163.com (X.C.); mumizhongfeng@126.com (F.Z.); wbgzu8307@gmail.com (B.W.); wangjh63@cau.edu.cn (J.W.); 2 Institute of Crop Sciences, Chinese Academy of Agricultural Sciences, No. 12 Zhongguancun South Street, Beijing 100081, China; 3 Institute of Nuclear Technology and Biotechnology, Xinjiang Academy of Agricultural Sciences, No. 403 Nanchang Road, Urumqi 830091, China; E-Mail: hquansheng@126.com

**Keywords:** CIPK, stress, salt tolerance, *Arabidopsis*

## Abstract

Salt stress represents an increasing threat to crop growth and yield in saline soil. In this study, we identified a maize calcineurin B-like protein-interacting protein kinase (CIPK), *ZmCIPK21*, which was primarily localized in the cytoplasm and the nucleus of cells and displayed enhanced expression under salt stress. Over-expression of *ZmCIPK21* in wild-type *Arabidopsis* plants increased their tolerance to salt, as supported by the longer root lengths and improved growth. The downstream stress-response genes, including dehydration-responsive element-binding (*DREB*) genes were also activated in transgenic plants over-expressing *ZmCIPK21*. In addition, introduction of the transgenic *ZmCIPK21* gene into the *Arabidopsis* mutant *cipk1-2* rescued the salt-sensitive phenotype under high salt stress. Measurement of Na^+^ and K^+^ content in transgenic plants showed that over-expression of *ZmCIPK21* decreased accumulation of Na^+^ and allowed retention of relatively high levels of K^+^, thereby enhancing plant tolerance to salt conditions.

## 1. Introduction

Soil salinity is one of the major abiotic stressors that negatively affect crop growth and yield. Over-abundance of salt induces ionic and osmotic stresses, which lead to secondary stresses, such as oxidative stress and nutritional disorders [1,2]. Plants can reduce cytoplasmic sodium levels through several mechanisms, including restriction of Na^+^ intake, increased excretion of Na^+^, and compartmentalization to vacuoles; they can also control the transport of Na^+^ from the roots to the aerial parts of the plant [3]. Maintaining a stable ratio of K^+^/Na^+^ in the cytoplasm is important for plant cell function because these ions regulate physiological and developmental processes [4].

Plants contain several groups of Ca^2+^-regulated proteins, including calmodulin, calcineurin B-like (CBL) protein, and calcium-dependent protein kinase (CDPK). CBL-interacting protein kinases (CIPKs) cooperate with CBL in multiple stress responses. In *Arabidopsis*, the Ca^2+^ sensor SOS3/CBL4 and its interacting kinase SOS2/CIPK24 are the well-characterized CBLs–CIPKs involved in salt tolerance. CIPK24 and CBL4 regulate the plasma membrane Na^+^/H^+^ antiporter SOS1, which contributes to plasma membrane salt regulation [5]. Activated SOS1 enhances salt detoxification via Na^+^ extrusion into the extracellular space [6]. Recent studies have revealed that the calcium sensor CBL10 is also able to activate CIPK24, which mediates sodium detoxification by modulating Na^+^ sequestration into the vacuolar compartment [7]. Similar to CIPK24, CIPK1 can form an alternative complex with either CBL1 or CBL9, and this complex mediates abscisic acid (ABA)-dependent and independent signaling responses. Interactions between CBL1 and CBL9 cause translocation of CIPK1 to the plasma membrane, and the loss of *CIPK1* in the *cipk1* mutant results in hypersensitivity to low concentrations of ABA and osmotic stress [8]. These data indicate the important role of the CBL–CIPK calcium-decoding system in plant signaling and adaptation reactions to salt stress.

In addition to protein kinases, transcription factors, and some stress-inducible genes also play important roles in regulating abiotic stress [9]. For example, the *RD29A*, *KIN1*, *COR15*, and *RAB18* genes are induced by several stresses. These genes can function to protect the cell structure, sustain protein expression and the cell membrane, and also assist in refolding denatured proteins [10]. Dehydration-responsive element-binding proteins (*DREBs*) are important plant transcription factors that regulate the expression of many stress-inducible genes, mostly in an ABA-independent manner, and play a critical role in improving the abiotic stress tolerance of plants. *DREBs* can bind to the *DREB* element to promote the expression of some stress-inducible genes [11]. Genetic modification of plants with *DREBs* may also contribute to the detoxifying effects of the protein and its downstream target protein [12].

Maize is an important crop throughout the world. Excessive salinity and drought have seriously affected maize yields in many regions. In the genome of the maize B73 inbred line, 43 putative *ZmCIPK* genes have been identified. Reverse-transcription polymerase chain reaction (RT-PCR) showed that *ZmCIPK* genes transcriptionally respond to many abiotic stresses, and some *ZmCIPK* genes are induced by salt, drought, heat, and cold stresses [13]. However, the functions of these maize *CIPK* genes have not been well studied. Here, we investigated the expression of the *ZmCIPK21* gene under abiotic stresses and found that over-expressing of *ZmCIPK21* in *Arabidopsis* up-regulated *DREB1B* and *DREB1C* genes and enhanced the salt tolerance of transgenic plants.

## 2. Results

### 2.1. Cloning and Phylogenetic Analysis of the ZmCIPK21 Gene

The full-length cDNA (Gene ID: GRMZM2G075002, NM_00154244) of a maize *CIPK* gene was found to share high similarity with *Arabidopsis CIPK1* and *CIPK21*; this gene was designated as *ZmCIPK21* (Figure S1). The *ZmCIPK21* gene was amplified from the cDNA of the inbred line B73, and the PCR product was cloned into the *pGWC-T* vector. Sequence analysis revealed that the open-reading frame (ORF) of this gene had a length of 1338 bp. To further investigate the phylogenetic relationships of *ZmCIPK21* and *CIPKs* of *Arabidopsis*, we performed neighbor-joining phylogenetic analysis using Clustal W and MEGA 4.1 software (The Biodesign Institute, Tempe, AZ, USA). Similar to other *Arabidopsis* *CIPKs,* *ZmCIPK21* contained two highly conserved domains, a kinase domain and an NAF domain. CIPK proteins can be divided into two subgroups based on their sequence similarities; Class I contain introns, and Class II do not contain introns [14]. *ZmCIPK21* contained 13 introns and thus belonged to the Class I subgroup of *CIPKs* (Figure S1). These data suggested the high evolutionary similarity between *ZmCIPK21* and *AtCIPK1* or *AtCIPK21*.

### 2.2. Over-Expression of ZmCIPK21 Enhanced Salt Tolerance in Arabidopsis

**Figure 1:**
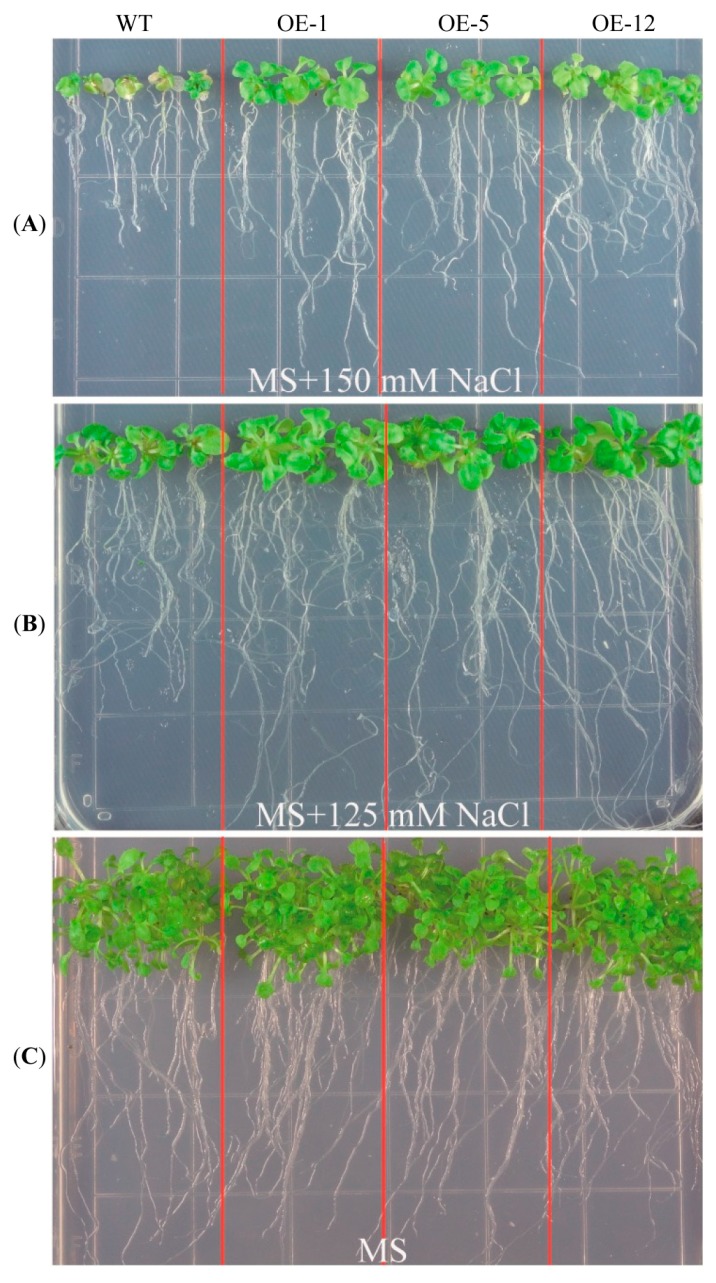
Performance of *ZmCIPK21*-over-expressing transgenic plants under salt stress. (**A**) Phenotypic comparison of transgenic (OE-1, OE5, and OE-12) and wild-type (WT) plants under 150 mM salt stress; (**B**) Transgenic and WT plants under 125 mM salt stress; (**C**) Transgenic and WT plants grown on Murashige and Skoog (MS) culture.

**Figure 2:**
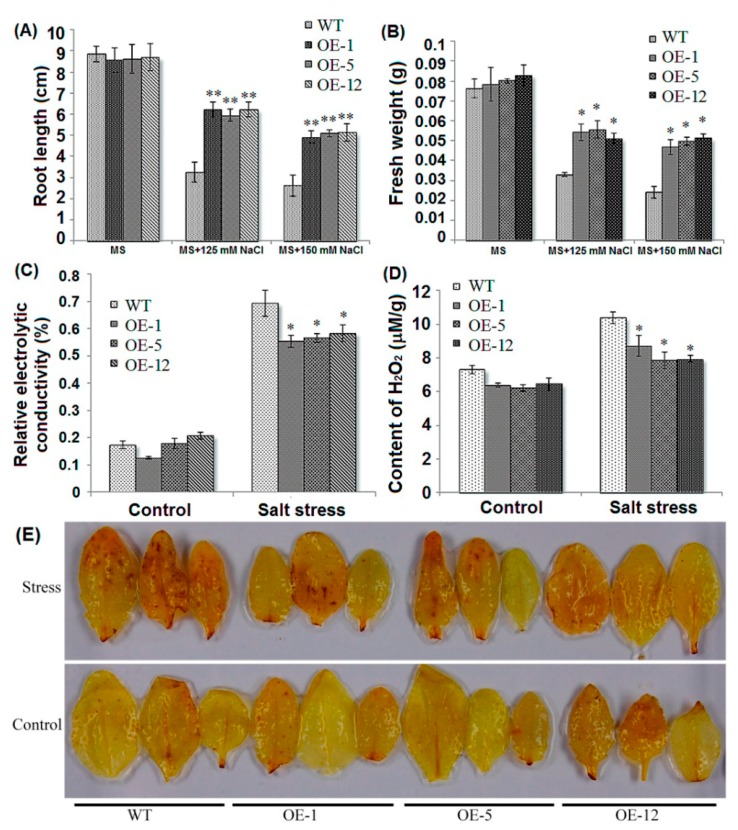
Physiological measurement of *ZmCIPK21*-over-expressing transgenic plants. (**A**) Root elongation of wild-type (Col-0) and *ZmCIPk21*-OE seedlings grown on MS agar plates containing different concentrations of NaCl (0, 125, or 150 mM) for 16 days; (**B**) Fresh weights of wild-type (Col-0) and *ZmCIPK21*-OE seedlings grown on MS agar plates containing different concentrations of NaCl (0, 125, or 150 mM) for 16 days; (**C**) Relative electric conductivity of leaves from *ZmCIPK21* OE and wild-type seedlings after salt stress (125 mM NaCl); (**D**) H_2_O_2_ accumulation in wild type and transgenic plants under control or salt stress (150 mM NaCl) conditions; (**E**) Diaminobenzidine (DAB) staining of true leaves of wild-type and transgenic plants under control or salt stress (150 mM NaCl) conditions. Asterisks indicate significant differences from the wild type at * *p* < 0.05 and ** *p* < 0.01.

Previously, we had reported that *ZmCIPK21* was up-regulated in response to salt stress [15]. Therefore, we generated transgenic *Arabidopsis* plants over-expressing *ZmCIPK21* and examined the growth of these transgenic plants under salt stress. In total, 11 transgenic lines were obtained, and *ZmCIPK21* transcripts in these plants were detected by RT-PCR analysis. ZmCIPK21-HA protein was also detected by western blotting using anti-HA antibodies (Figure S2), and three independent T_3_ homozygous lines (named OE-1, OE-5, and OE-12) were selected for subsequent studies. After treatment with 125 or 150 mM NaCl, the *35S:* *ZmCIPK21* transgenic plants showed markedly higher salt tolerance than wild-type plants (Figure 1A,B). The root length of the transgenic plants was twice that seen in the wild-type plants following treatment with 150 mM NaCl (Figure 2A). There were also significant differences in the fresh weights of wild-type and transgenic plants, the fresh weights of transgenic plants were higher than those of the wild-type plants under salt stress (Figure 2B). No significant differences were observed between the wild-type and transgenic lines under normal growth conditions (Figure 2A,B).

Furthermore, we also measured the relative electric conductivity of the leaves in transgenic and wild type plants. Ten-day-old seedlings were exposed to 125 mM NaCl, and the relative conductance was determined 24 h later. Statistical analysis indicated that transgenic plants exhibited significantly lower relative conductance rates than wild-type plants. In contrast, there was no difference under normal growth conditions (Figure 2C). Salt stress causes massive generation of reactive oxygen species (ROS), which can interfere with metabolism [16]. Therefore, the generation of H_2_O_2_ in transgenic and wild-type plants was measured. We found that wild-type and transgenic plants exhibited increased levels of H_2_O_2_ following exposure to 150 mM NaCl. As expected, the content of H_2_O_2_ was higher in wild-type plants than in transgenic plants, and no differences were observed under normal growth conditions (Figure 2D,E). These results suggested that over-expression of *ZmCIPK21* might enhance membrane stability and maintain low levels of ROS under salt stress.

### 2.3. Over-Expressing of ZmCIPK21 Resulted in Up-Regulation of Two DREB Genes in Arabidopsis

**Figure 3:**
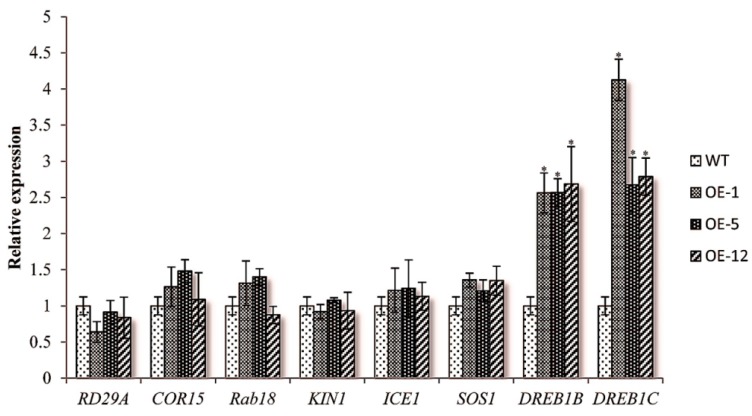
Expression patterns of stress-related marker genes in *ZmCIPK21*-OE and wild-type seedlings. Plants were grown for 16 days under normal conditions, and RNA was isolated from each sample. Gene expression was assayed by quantitative reverse transcriptase-polymerase chain reaction (qRT-PCR) with gene-specific primer pairs. The *Arabidopsis* actin gene was used as a reference. The 2^−∆∆*C*t^ method was used to measure the relative expression level of the target gene. Expression values in transgenic plants were compared with those in wild-type plants. The experiments were performed in triplicate, and values are the means ± SE of three samples. Asterisks indicate significant differences from the wild type at * *p* < 0.05.

To investigate how over-expression of *ZmCIPK21* affected downstream genes in response to abiotic stress, we analyzed the expression of several marker genes in transgene plants under normal conditions, including *RD29A*, *KIN1*, *RAB18*, *COR15*, *Rab18*, and *ICE*, which have been shown to be responsive to multiple stress signals, such as osmotic stress and ABA treatment [17,18,19]. In addition, we evaluated the expression of *DREB1B* and *DREB1C*, transcription factors that are expressed early in the abiotic stress response [20,21]. The expression pattern of *DREB1C* was similar to that of *DREB1B*, which was dramatically induced in *ZmCIPK21*-ove-rexpressing plants, while the expression levels of *SOS1*, *COR15*, *Rab18*, and *ICE* were not significantly different between transgenic and wild-type plants. The expression levels of *RD29A* and *KIN1* in OE-1 and OE-12 were slightly decreased, but there were no significant differences between the three transgenic lines (Figure 3). Therefore, our results suggested that increased expression of *DREB1B* and *DREB1C* might contribute to the production of transgenic plants with enhanced salt tolerance.

### 2.4. Subcellular Localization of ZmCIPK21

**Figure 4:**
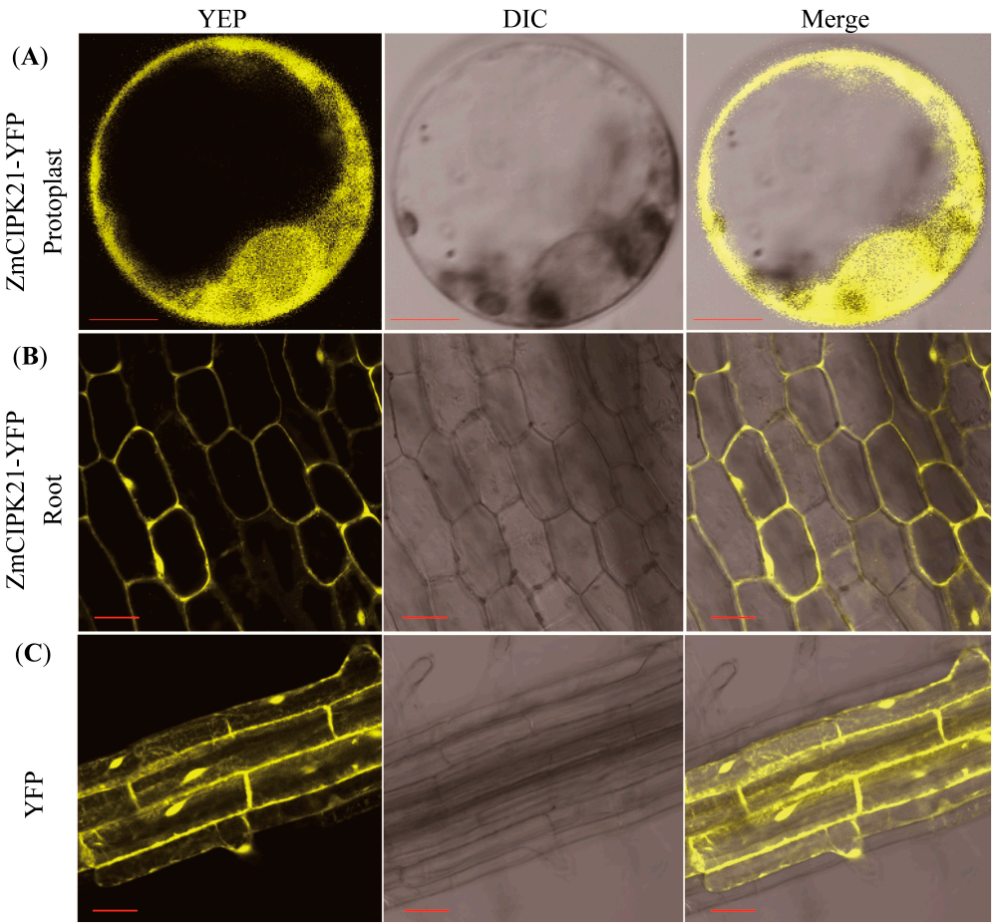
Localization of *ZmCIPK21* in *Arabidopsis* roots and protoplast of leaves. (**A**) The protoplasts of the *35S:ZMCIPK21-YFP* plants; (**B**) The *35S:ZmCIPK21-YFP* plants roots expressing *YFP*-fused *ZmCIPK21*; (**C**) *Arabidopsis* roots expressing *35S:YFP*. 200× magnification. Scale bar = 10 μm.

To investigate the localization of *ZmCIPK21*, the *35S:ZmCIPK21-YFP* plasmid was constructed and transformed into *Arabidopsis* by the floral dip method. Homozygous transgenic lines were used for localization analysis. First, we analyzed the localization of the *ZmCIPK21-YFP* fusion protein in roots, in the root tip, we observed fluorescence representing *ZmCIPK21-YFP*, which was primarily localized in the cytoplasm and nucleus of the root cells from 6-day-old seedlings. Next, we collected the protoplast by digesting the cell walls of *35S:ZmCIPK21-YFP* plant leaves. Using confocal microscopy, we found that *ZmCIPK21-YFP* was primarily localized in the cytoplasm and nucleus. In contrast, fluorescent signals were found throughout the cells when *YFP* was expressed alone (Figure 4).

### 2.5. ZmCIPK21 Rescued the Sensitivity of the Arabidopsis Cipk1-2 Mutant

**Figure 5:**
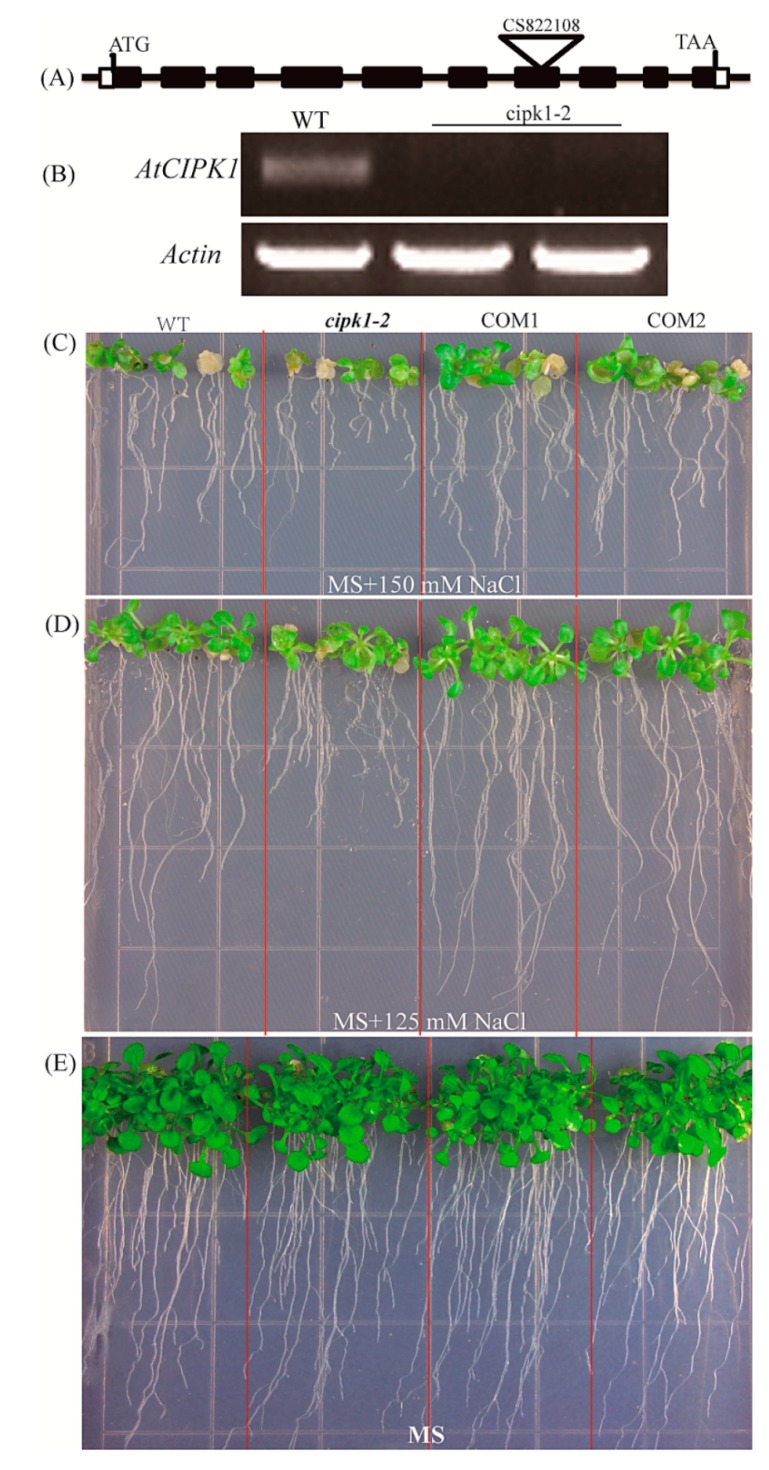
The salt-sensitive phenotype of *Arabidopsis* *cipk1-2* was complemented by over-expressing of ZmCIPK21. (**A**) Intron-exon structure of the *Arabidopsis* *CIPK1* gene and location of the T-DNA insertion; (**B**) Transcript levels of *CIPK1* in the *Arabidopsis* *cipk1-2* mutant; (**C**–**E**) Phenotypes of wild-type, *cipk1-2*, and *ZmCIPK21* in *cipk1-2* (COM) lines grown on MS agar plates containing different concentrations of NaCl (0, 125, or 150 mM).

The sequence of *ZmCIPK21* had 61% amino acid similarity to *Arabidopsis CIPK1* (Figure S1), and we hypothesized that *ZmCIPK21* may share a conserved function with the *Arabidopsis CIPK1* gene. In this study, we identified an *Arabidopsis* *cipk1* mutant (CS822108) from ABRC stocks where a T-DNA was inserted in the seventh exon; we named this mutant *cipk1-2*. The *cipk1-2* mutation was analyzed by PCR using a *CIPK1*-specific primer and a T-DNA left border primer. Absence of the *CIPK1* transcript in *cipk1-2* was confirmed by RT-PCR analysis with gene-specific primer pairs (Forward: 5'-GGAGACCCACCAATACCAAG-3', Reverse: 5'-GATCTACGCCACGGTTTATACTT-3') in the homozygous mutant (Figure 5B). When 5-day-old mutant and wild-type (Col-0) plants were transferred to MS medium containing 150 mM NaCl, 0.3 μM ABA, and 325 mM mannitol, the *cipk1-2* mutant showed higher sensitivity than the wild-type plants (data not shown).

To examine whether *ZmCIPK21* could rescue the *cipk1-2* phenotype, the *ZmCIPK21* gene driven by the *35S* *CaMV* promoter was transformed into *Arabidopsis* *cipk1-2* mutant plants, and homozygous transgenic lines were obtained and used for analysis. The salt tolerance of transgenic *ZmCIPK21* in *cipk1-2* complementation (COM) plants was investigated at the seedling stage. After 5 days of germination on normal MS media, the seedlings were transferred to MS media plates with 125 or 150 mM NaCl. After 14 days, the COM lines exhibited increased root growth compared to the *cipk1-2* mutant under conditions of high salt stress, similar to the wild-type seedlings (Figure 5C,D). The root lengths of COM and wild-type plants were significantly different from those of the *cipk1-2* mutant when grown with 150 mM NaCl. On normal MS media, no significant differences were observed among the COM seedlings, *cipk1-2* mutant seedlings, and wild-type seedlings (Figure 5E). These results suggested that *ZmCIPK21* could complement the salt tolerance function of *AtCIPK1* in the *cipk1-2* mutant.

### 2.6. Over-Expressing of ZmCIPK21 Maintained Na^+^ and K^+^ Homeostasis in Plant Cells

**Figure 6:**
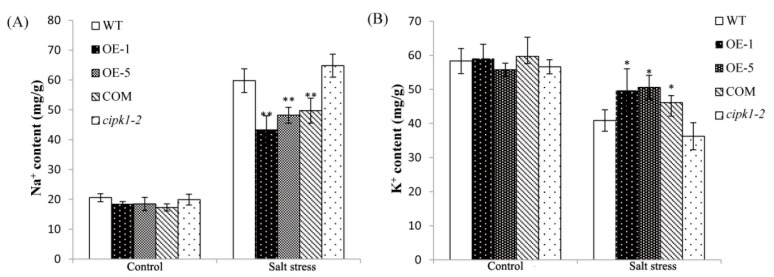
Na^+^ and K^+^ accumulation in transgenic and control plants. (**A**) Na^+^ concentration in wild-type, *cipk1-2*, *ZmCIPK21-OE*, and complementary (COM) seedlings under control and stress conditions after 6 days; (**B**) K^+^ concentration in wild-type, *cipk1-2*, *ZmCIPK21-OE*, and COM seedlings under control and stress conditions after 6 days. Data were obtained from three independent experiments and are presented as the mean ± SE. Asterisks indicate significant differences from the wild type at * *p* < 0.05 and ** *p* < 0.01.

Accumulation of sodium causes changes in potassium ion levels. The maintenance of K^+^/Na^+^ homeostasis is an important requirement for salt tolerance [22]. To test whether over-expressing of *ZmCIPK21* reduced sodium accumulation in plants, the Na^+^ and K^+^ contents of transgenic plants and the *cipk1-2* mutant were tested. After salt stress, *ZmCIPK21*-over-expressing lines and COM lines accumulated less Na^+^ than wild-type and *cipk1-2* mutant lines. The Na^+^ content of OE plants was about 43 mg/g, In contrast, wild-type plants and the *cipk1-2* mutant exhibited significant increases in Na^+^ ion contents up to 60 mg/g. There were no differences between OE plants and COM plants (Figure 6A). Relative to Na^+^, the K^+^ content in *ZmCIPK21*-over-expressing lines and COM plants exhibited relatively smaller changes than those of the *cipk1-2* mutant and wild-type plants following salt treatment, and the K^+^ content in OE plants and COM plants decreased slightly from 60 to 50 mg/g. This was in contrast with wild-type plants and *cipk1-2* mutants, in which the K^+^ content decreased to 40 and 36 mg/g, respectively (Figure 6B). Under normal conditions, the Na^+^ and K^+^ contents of transgenic plants and the COM line did not differ from those of wild-type and mutant plants.

## 3. Discussion

Although many *CIPK* genes have been identified, the functions of *CIPKs* have been studied mainly in *Arabidopsis*. The precise role of CIPKs in salt stress tolerance is not completely understood, especially for maize. Here, we identified a novel maize *CIPK* gene, designated *ZmCIPK21*, and discovered that *ZmCIPK21* improved salt tolerance in transgenic *Arabidopsis* plants.

*ZmCIPK21* shares a high similarity with other plant *CIPK* family members in terms of sequence motif analysis. Generally, a *CIPK* protein has two conserved domains, the *N*-terminal kinase domain and the *C*-terminal regulatory domain. There are additional putative domains in *ZmCIPK21*, including an activation loop with a threonine residue starting with the conserved DFG motif and ending with the APE motif, which is targeted for phosphorylation by protein kinases, and the junction domain, which is further responsible for kinase activation (Figure S1).

Phylogenetic tree analysis showed that *ZmCIPK21* had a high sequence similarity to *Arabidopsis CIPK1*, *CIPK21*, *CIPK17*, *CIPK23*, *CIPK24*, and other *CIPKs* belonging to the intron-rich clade. Motif domain analysis, together with the phylogenetic tree analysis presented here, will facilitate the functional annotation and study of *ZmCIPKs*. When *35S:ZmCIPK21* was transformed into the *Arabidopsis cipk1-2* mutant, the salt-sensitive phenotype was restored to wild-type levels. In recent years, much has been learned about how the *CBL-CIPK* network is involved in stress signaling, and many of the details through which this network participates in crosstalk in various plants have been elucidated. [13,23]. Genetic analysis showed that species-specific duplication or deletion events contributed to the numerical divergence of the *CIPK* gene family. Segmental duplication explains a greater proportion of the current complexity of the *CIPK* gene family. Comparing the maize *CIPKs* gene family to *Arabidopsis* and rice *CIPKs*, it has been found that they share high colinearity with each other [23,24]. Therefore, *ZmCIPK21* may be sharing some conserved signal transmission mechanisms with *CIPK1* in *Arabidopsis* to salt tolerance.

In the maize genome of the B73 line, there are at least 43 putative *CIPK* genes, and many *ZmCIPKs* can be induced by one or more abiotic stresses [13]. Previously, we had reported that *ZmCIPK21* transcripts were significantly induced by salt treatment. However, under ABA treatment, *ZmCIPK21* was only slightly up-regulated. Under abiotic stresses, many CBLs recruit CIPKs to the plasma membrane and regulate various targets, which have a function in ion homeostasis [25]. The mechanism of salt tolerance includes restriction of Na^+^ intake, increased excretion of Na^+^, and compartmentalization to vacuoles; they can also control the transport of Na^+^ from the roots to the aerial parts of the plant [3]. In this study, transgenic plants accumulated less Na^+^ than wild-type plants, and there were significant differences in the fresh weights and root lengths between transgenic plants and wild-type plants after salt stress. Therefore, we speculated that *ZmCIPK21* over-expression might ensure proper performance of physiological activities by lowering levels of Na^+^ accumulation. Furthermore, the relative electrical conductivity and ROS generation of transgenic plants were reduced compared with those of wild-type plants. These results implied that the salt tolerance of plants over-expressing *ZmCIPK21* might function to restrict Na^+^ absorption in roots.

Accumulation of sodium causes imbalances in potassium ion levels that affect biochemical reactions [26]. The root surface absorbs the mineral elements in the soil and selectively guides tube transport processes. Under high concentrations of sodium ions, absorbtion of potassium ions will be inhibited through a somehow competitive inhibition for the K^+^ transport system [27,28]. In this study, *ZmCIPK21*-over-expressing lines accumulated less Na^+^ than wild-type and *cipk1-2* mutant lines, and the K^+^ content in transgenic plants and *ZmCIPK21* in *cipk1-2* COM lines decreased less than those of wild-type and *cipk1* mutant plants under salt stress. A previous study reported that *Arabidopsis* *CIPK1* interacts with CBL9 and that CBL1 targets the kinase to the plasma membrane. Accordingly, mutations in either CBL1 or CIPK1 render the plants hypersensitive to osmotic stress [8]. In this study, we analyzed the osmotic stress of OE plants, but there are no significant differences between OE lines and wild-type plants (data not shown). It is increasingly evident that multiple stress perception and overlapping signal transduction pathways exist in plants, which may exhibit crosstalk at various steps. In this study, the salt tolerance of COM lines was rescued to levels of those observed for the wild-type, and the K^+^ content of *ZmCIPK21*-over-expressing lines and COM plants exhibited relatively smaller changes than those in the *cipk1-2* mutant and wild-type plants following salt treatment. The aerial parts of COM plants appeared to be larger than wild-type plants. One explanation may be that the protein kinase *CIPK1* does not serve a predominant role in mediating plant responses to salt stress, and there may be many other cofactors involved in *ZmCIPK21*-dependent maintenance of Na^+^/K^+^ homeostasis under salt stress.

Transcription factors typically regulate the expression of multiple genes in a metabolic pathway and are known to play an essential role in stress responses. In this study, we found that the expression levels of *DREB1B* and *DREB1C* were higher in *ZmCIPK21*-over-expressing plants than in wild-type plants. Although the mechanism mediating the high expression of *DREB1B and DREB1C* in the transgenic plants is unknown, it is clear that high expression of the *DREB1* gene would affect plant tolerance to abiotic stress by regulation of the expression of some downstream genes. The essential role of the *DREB1* gene in response to cold, drought, and salt stress has been demonstrated in previous studies [29,30]. Over-expression of *DREB1* by the 35S *CaMV* promoter increases stress tolerance to drought, high salinity, and freezing in transgenic *Arabidopsis* plants [31,32]. The ectopic over-expression of *Solanum DREB1* confers increased tolerance to high-salt conditions [33]. These results suggest that *ZmCIPK21* may play a positive role in salt stress by regulating *DREB1B* and *DREB1C* in *Arabidopsis*.

## 4. Experimental Section

### 4.1. Arabidopsis Growth and Analysis of Salt Tolerance

*Arabidopsis thaliana* Col-0 was used as the wild-type line in this experiment. The homozygous *cipk1-2* line was identified from a SALK line (CS822108) with a T-DNA insertion in the seventh exon of At3G17510 and was purchased from *Arabidopsis* Biological Resource Center Stocks (Columbus, OH, USA)*.* Seeds were surface-sterilized with 10% sodium hypochlorite for 10 min and extensively washed five times with sterile water. Sterile seeds were planted on MS medium plates with 0.8% agar and incubated at 4 °C for 3 days. For salt tolerance assays on plates, wild-type, *cipk1-2*, and COM lines were grown vertically on MS medium with 0.8% agar for 5 days at 23 °C. The seedlings were then transferred to MS medium without or with 125 or 150 mM NaCl. After the indicated times of vertical growth, seedlings were photographed and root lengths and fresh weights were measured. The H_2_O_2_ content in the tissues was measured according to the method of Gay and Gebicki [34]. All experiments were performed in triplicate.

### 4.2. Generation of Transgenic Plants Over-Expressing the ZmCIPK21 Gene

The *ZmCIPK21* coding region (1337 bp) was amplified using the following primers: forward, 5'-ATGCGGATGGGCAAGTACGA-3' and reverse with an HA tag, 5'-TTAAGCGTAATCTGGAACATCGTATGGGTAAAAGCTACTATTATATCTAG-3'. The PCR product was cloned into the *pGWC-T* vector and then into the binary 35S *CaMV* over-expressing vector *pEarlyGate100* using Gateway technology [35]. The resulting plasmid was introduced into the *Agrobacterium tumefaciens* strain GV3101 and transformed into *Arabidopsis* by the floral dip procedure [36]. Transgenic lines expressing high levels of *ZmCIPK21* were propagated for three generations and screened by spraying with 0.05% (*v*/*v*) phosphinothricin (PPT).

### 4.3. Quantitative PCR Analysis of Gene Expression in Arabidopsis

Total RNA was extracted from seedling leaves using TRIzol (Invitrogen, Carlsbad, CA, USA) according to the manufacturer’s instructions. RNA was treated with DNase (New England Biolabs Inc., Beverly, MA, USA) according to the manufacturer’s instructions. cDNA was then synthesized from 300 ng of RNA using SuperScript III First-Strand Synthesis SuperMix (Invitrogen, Carlsbad, CA, USA). Real-time quantitative PCR analysis was performed with a 7300 sequence detection system (Applied Biosystems, Foster City, CA, USA). The amounts of template cDNA that were used in each PCR reaction were corrected by the results of quantification of the *actin* gene. The primers used are presented in Table 1. Data were processed using the 2^−∆∆*C*t^ method to calculate gene expression in response to changes in stress treatment [37].

**Table 1:** Primers used for quantitative PCR analysis.

Gene	Upstream Primer (5'-3')	Downstream Primer (5'-3')
*RD29A*	CCCGGATCCTTTTCTGATATGGTTGCC	GCCCTCGAGCCGAACAATTTATTAACC
*COR15*	AACTCTGCCGCCTTGTTTGC	CTTGGTGCAAGTGCTGTGAT
*Rab18*	AGCAGCTTGCTTCTCAGCTT	ATTCCTTCTTCCCGGCTAACCT
*KIN1*	TGCCTTCCAAGCCGGTCAGA	AGGCCGGTCTTGTCCTTCAC
*ICE1*	GGAGATGCAATTGATTATCTGAAG	AGTCAGGATCCGATCAGATCATACC
*SOS1*	GACGGGAGAATCAATCGAAA	TGCTCTTGCTCTCGTCTCAA
*DREB1B*	GGCGTTGGCTTTTCAAGATG	AAGTCGGCATCCCAAACATT
*DREB1C*	TTCGATTTTTATTTCCATTTTTGG	CCAAACGTCCTTGAGTCTTGAT
*Actin*	TAACCCAAAGGCAACAGAG	CTTGGTGCAAGTGCTGTGAT
*ZmCIPK21*	CAGAGGGTGGCAAGAAGGAATTGT	GTTGCTTCAGTCCATTTCCFGTACC
*ZmGAPDH*	GGGTGAGGCTGGTGCTGAGTATGT	TTAGCAAGGGGAGCAAGGCAGTT

### 4.4. Subcellular Localization

For analysis of ZmCIPK21 localization, the coding sequence of *ZmCIPK21* was cloned into the *pEarlyGate101* vector to create a fusion construct with the *C*-terminal fragment of yellow fluorescent protein (YFP) [38]. *Arabidopsis* plants were transformed by Agrobacterium-mediated floral dip. After three generations of selection, homozygous transgenic lines were obtained. The protoplasts were isolated from leaves as follows. The leaves were cut into pieces and quickly transferred in to 0.6 M mannitol. Then the mannitol solution was discarded and replaced with a digestion solution containing 0.5 M mannitol, 10 mM 4-morpholineethanesulfonic acid (MES), 1.5% cellulose Onozuka RS (Yakult, Tokyo, Japan), 0.75% macerozyme Onzuka R-10, 10 mM CaCl_2_, and 0.1% bovine serum albumin (BSA), pH 5.7. The samples were incubated for 3 h in the dark with gentle shaking (40–50 rpm) at 28 °C. The digestion solution was then shaken to release the protoplasts followed by filtration though a 50 µm cell strainer to collect the protoplast suspension. Fluorescence analysis was performed on a LEICA TCS SP2 confocal laser scanning system (Leica Microsystems, Wetzlar, Germany). YFP fluorescence was examined at 514 nm (excitation) using an argon laser with an emission band of 515–530 nm.

### 4.5. Measurement of Na^+^ and K^+^ Content

For measurement of Na^+^ and K^+^ content in plant tissues, the seedlings of wild-type and transgenic *Arabidopsis* plants and COM lines were planted in a medium (soil:vermiculite = 4:6) under light/dark cycle conditions of 16/8 h at 22 °C. After 14 days, seedlings were watered with 100 mM NaCl solution for 6 days. Leaves from the rosette were excised carefully to determine their Na^+^ and K^+^ content. After 24 h at 105 °C, the dry weight was measured. The resulting dry matter was dissolved in nitric and perchloric acid (4:1) on a muffle furnace at 175 °C for 3 h. When the liquid became limpid, the Na^+^ and K^+^ contents of the samples were determined with an atomic absorption spectrophotometer [39].

## 5. Conclusions

In conclusion, over-expression of *ZmCIPK21* in *Arabidopsis* caused high expression of *DREB1B* and *DREB1C* and enhanced the salt tolerance of *Arabidopsis* by maintaining Na^+^ homeostasis. Therefore, *ZmCIPK21* can be used as a candidate gene to improve stress tolerance by genetic transformation in crops.

## Supplementary Files

Supplementary File 1
