# Supplementary material for: ZmCIPK21, A Maize CBL-Interacting Kinase, Enhances Salt Stress Tolerance in Arabidopsis thaliana
Source: Int J Mol Sci. 2014 Aug 22;15(8):14819–34. doi: 10.3390/ijms150814819 (PMC4159884; doi:10.3390/ijms150814819)

Supplementary Information

Figure S1. Sequence analysis of *ZmCIPK21*. (**A**) Comparison of five class II CIPK amino acid sequences. The black boxes indicate identical residues, and gray boxes indicate residues conserved among the proteins; (**B**) Phylogenetic relationship of ZmCIPK21 and *Arabidopsis* CIPKs. Multiple sequence alignment was performed using MEGA4.1, and the phylogenetic tree was constructed following the neighbor-joining method using the amino acid sequence of each CIPK protein. The accession numbers of selected CIPKs are listed as follows: ZmCIPK21, GRMZM2G075002, NM_00154244; AtCIPK1, NM_112631; AtCIPK21, NM_125144; AtCIPK24, NM_125144; AtCIPK23, NM_102766; AtCIPK3, NM_128256; AtCIPK9, NM_099996; AtCIPK8, NM_118573; AtCIPK17, NM_103723.

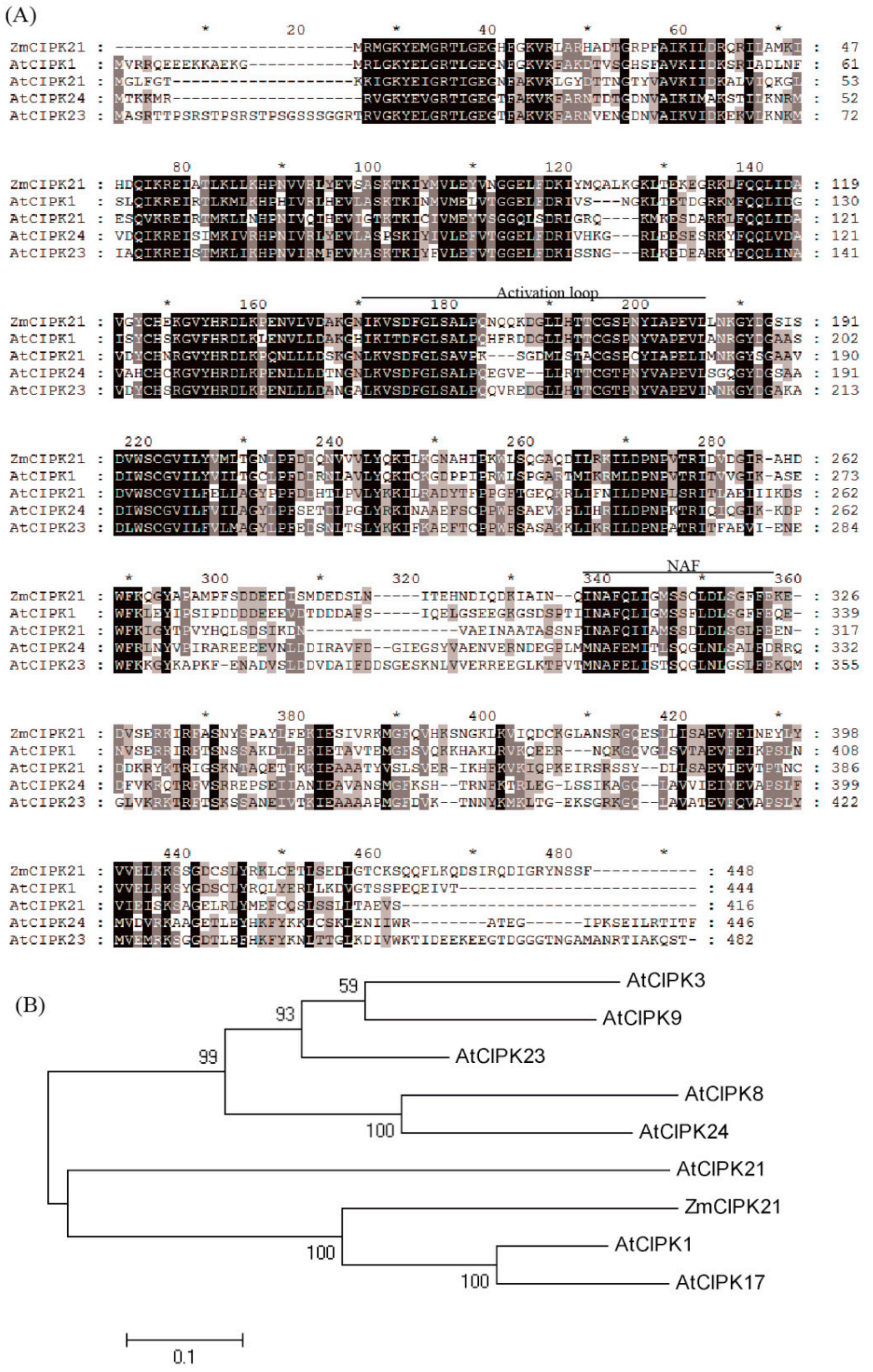

Figure S2. Expression of *ZmCIPK* in *ZmCIPK*-OE plants. The upper panel shows the RT-PCR analysis of 11 *ZmCIPK*-OE plants, with *actin* as an internal reference. Western blots of ZmCIPK-HA proteins are shown in the lower panels.

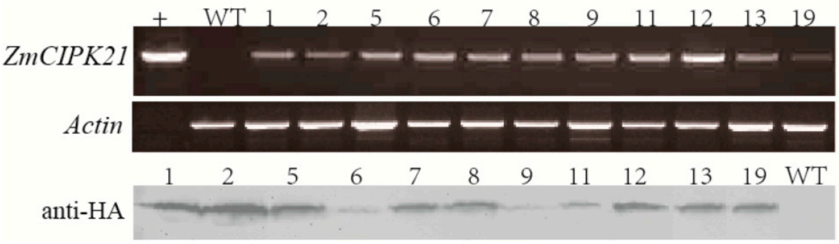

Supplement: Supplementary File 1 [file ijms-15-14819-s001.pdf]
